# Whoop! There it is: The surprising resurgence of pertussis

**DOI:** 10.1371/journal.ppat.1008625

**Published:** 2020-07-23

**Authors:** Audra R. Fullen, Kacy S. Yount, Purnima Dubey, Rajendar Deora

**Affiliations:** 1 Department of Microbial Infection and Immunity, The Ohio State University, Columbus, Ohio, United States of America; 2 Comprehensive Cancer Center, The Ohio State University, Columbus, Ohio, United States of America; 3 Department of Microbiology, The Ohio State University, Columbus, Ohio, United States of America; University of Massachusetts, Worcester, UNITED STATES

Despite high global vaccine coverage, whooping cough, also known as pertussis, caused by the gram-negative obligate human pathogen *Bordetella pertussis (Bp)*, is resurging worldwide. The inactivated whole cell vaccines (wPV), introduced in the 1940s, were extremely effective in preventing severe disease, controlling the bacterial burden in the entire respiratory tract, and preventing transmission. Because of severe reactogenicity and negative public perceptions regarding safety, wPV were discontinued and acellular subunit (1 to 5 protein components) vaccines adjuvanted with alum (aPV) were introduced in many countries. While safer and effective in disease prevention, these aPVs elicit poor and short-lived immunity and fail to prevent infection [1, 2]. Here, we discuss reasons for pertussis resurgence, bacterial evolution, and limitations of current pertussis vaccines. We also propose new directions to fill existing research gaps and accelerate the development of more effective vaccines.

## Genotypic and phenotypic variations between vaccine reference and circulating strains of *Bp*

*Bp* research and vaccine formulations are largely based on Tohama I, a strain isolated in Japan in the 1950s and its derivatives. Recent whole genome sequencing and chromosomal analyses of circulating *Bp* (*cBp*) strains changed the prevailing view of *Bp* as a monomorphic pathogen with small genetic changes and SNPs. Instead, *cBp* strains represent a dynamic population whose genomes exhibit extensive structural rearrangements including large inversions, duplications, and deletions [3, 4]. Nonetheless, comparative genomics alone cannot sufficiently explain pertussis resurgence. Going forward, it is important to understand how genomic variations in *cBp* strains lead to alterations in phenotypic and pathogenic properties.

Few studies have examined phenotypic differences between the reference and *cBp* strains. Variations in the amounts of aPV antigens pertactin (Prn), pertussis toxin (PT), filamentous hemagglutinin (Fha), and fimbriae (Fim2/3) have been observed. Compared to reference strains, *cBp* strains from several countries isolated subsequent to aPV introduction either fail to produce or produce higher amounts of Prn, Fha, and PT. Strain-specific differences in production of three Fim serotypes have also been observed. These results have led to the hypothesis that due to aPV-induced selection pressure, divergence in aPV antigens is observed at higher rates than in factors not included in aPV [3, 5]. However, whether these genotypic and phenotypic differences result in the reduction of aPV efficacy remains controversial.

With respect to non-aPV factors, some *cBp* strains are less susceptible to complement-mediated killing, a phenotype linked to strain-dependent variations in the expression of Vag8 [6]. *Bp* reference strains also do not produce the Type III secretion system effector proteins, which are produced in low-passaged and *cBp* strains [7]. Continued genotypic and phenotypic evaluation of *cBp* strains and investigation of the mechanisms by which these strains resist and modulate host innate and adaptive immune responses is necessary to comprehensively understand pertussis resurgence. To combat future antigenic divergence, it is important to identify highly conserved and essential antigens produced by *cBp* strains from multiple countries and include them in next-generation aPV.

## Determinants of *Bp* nasal colonization, persistence, and transmission

The mechanisms by which *Bp* persists in the nasopharynx and transmits between humans is poorly studied. *Bp* forms multicellular aggregates and biofilms on the mouse nasal septum and trachea, which resemble structures present on human tissue explants and respiratory tissues of patients [9]. Compared to reference strains, *cBp* strains isolated from multiple countries exhibit increased autoaggregation and biofilm formation [9, 10]. These phenotypes are correlated with increased expression and production of biofilm-promoting factors Fha and *Bordetella* polysaccharide (Bps) and decreased or negligible activity of the biofilm-inhibiting factor adenylate cyclase toxin [10]. Importantly, these strains exhibit increased epithelial cell adherence and bacterial burden on the mouse nasal septum and trachea [10]. Thus, it is likely that *cBp* strains are evolving to acquire enhanced virulence. We postulate that *Bp* biofilms are protected from killing by host immune components and thus promote establishment of a chronic carrier state in the nasopharynx.

Airborne particles or respiratory droplets are the principal means of *Bp* transmission. The impact of particle size on *Bp* transmission is not known. In general, particles less than 10 μm in diameter penetrate deeper into the respiratory tract whereas particles equal to or greater than 10 μm in diameter are deposited onto upper airway surfaces and penetrate poorly into lower pulmonary regions [11]. *Bp* is relatively small (0.4 to 0.8 μm). Thus, formation of differently sized aerosol particles or droplets either by autoaggregation or by dispersal of established biofilms can result in differences in the extent of transmissibility and infectivity of *cBp* strains, an area that requires further investigation.

A major limitation of aPV is the absence of an antigen with a proven role in colonization of the upper respiratory tract. Bps is the first known factor to promote attachment and efficient colonization of *Bp* in the mouse nose. It is also essential for biofilm growth and maturation on the mouse nasal septum [8]. Thus, conceptually, a Bps-containing aPV could control bacterial colonization and subsequent transmission.

## Animal models of immunization and *Bp* infection

Mice serve as excellent models to investigate *Bp* pathogenesis and vaccine efficacy since they display many parallels to human infections. While mice do not display the human symptoms of cough and leukocytosis, these differences do not significantly affect the aforementioned phenotypic evaluations. However, adult mice do not transmit bacteria [12]. Recently, *Bp* transmission between neonatal mice was reported [13]. Infection of neonatal mice also recapitulates many aspects of pertussis including the more severe and sometimes fatal disease occurring in human infants [13]. Larger animal models utilized to study *Bp* include pigs and baboons [12, 14]. Infant and adult baboons infected with *Bp* experience leukocytosis, cough, and transmit bacteria. While wPV immunization efficiently reduces bacterial burden in the entire respiratory tract of mice and baboons, aPV immunization of mice and baboons clears only the lower respiratory tract. Additionally immunization with wPV but not with aPV prevents transmission in baboons, confirming long-standing epidemiological data in humans [14]. Continued utilization of these animal models will provide insights into the pathogenic consequences of genomic and phenotypic variations in *cBp* strains. Additionally, the neonatal mouse model will allow testing of next-generation maternal vaccines, while adult mice and baboons will continue to be useful for testing vaccines for infants through adults.

## Failure of aPV to induce an optimal immune response

wPV and natural *Bp* infection induce optimal and long-lived T helper 1 and T helper 17 (Th1/17)-polarized cellular and humoral immune responses, while aPV primarily elicit Th1/2-polarized and short-duration immune responses. A vigorous ongoing research area is the development and validation of next-generation aPV that elicit Th1/17-polarized responses similar to those induced by wPV and natural infection. These experimental aPV include formulations that contain toll-like receptor 2 (TLR2), TLR9, and STING (stimulator of interferon genes) /cGAS (cyclic GMP-AMP synthase) ligands as adjuvants [15]. A live attenuated strain, BPZE1, genetically engineered to remove three *Bp* toxins, reduced bacterial numbers in the nose, elicited Th1/17 responses and was found to be safe for healthy adults in a Phase I clinical trial [16]. Intranasal delivery of these experimental subunit vaccines or BPZE1 protected mice from *Bp* challenge at 10 months postimmunization [15, 17]. The long-lived protection was obtained by intranasal but not systemic immunization with these vaccines, suggesting that the mucosal immunization route is more protective than the current regimen of intramuscular vaccine delivery. Natural infection and wPV vaccination induces robust CD4^+^ tissue-resident memory T cells (T_RM_) in the respiratory tract that are critical for bacterial clearance, while alum-adjuvanted aPV do not. Intranasal immunization of mice with BPZE1 or an aPV with a Th1/17-inducing combination adjuvant elicits T_RM_ production in the nose and lungs [17, 18].

An important unanswered question is whether aPV-induced protection can be improved and extended for individuals previously immunized with currently commercialized aPV, which includes Th2-skewing alum as the adjuvant. Bordetella Colonization Factor A (BcfA) has been shown to have Th1/17-skewing adjuvant properties. Addition of BcfA to a commercial aPV attenuated the Th2 responses primed by alum and accelerated clearance of *Bp* from mouse lungs [19], suggesting that a BcfA-containing vaccine may improve the longevity of protection. Thus, a modified vaccine that replaces alum or in combination with a Th1/17-inducing adjuvant delivered intranasally may be the key to long-lived protection and reduced transmission.

Current aPV antigens were selected primarily for their ability to induce antibody responses, which, while contributing to bacterial clearance, may not be sufficient or essential. Improving next-generation aPV will also require identification of CD4^+^ T cell epitopes by bioinformatics and proteomics combined with mass spectrometry [20].

## Conclusion and multipronged future directions

**Fig 1:**
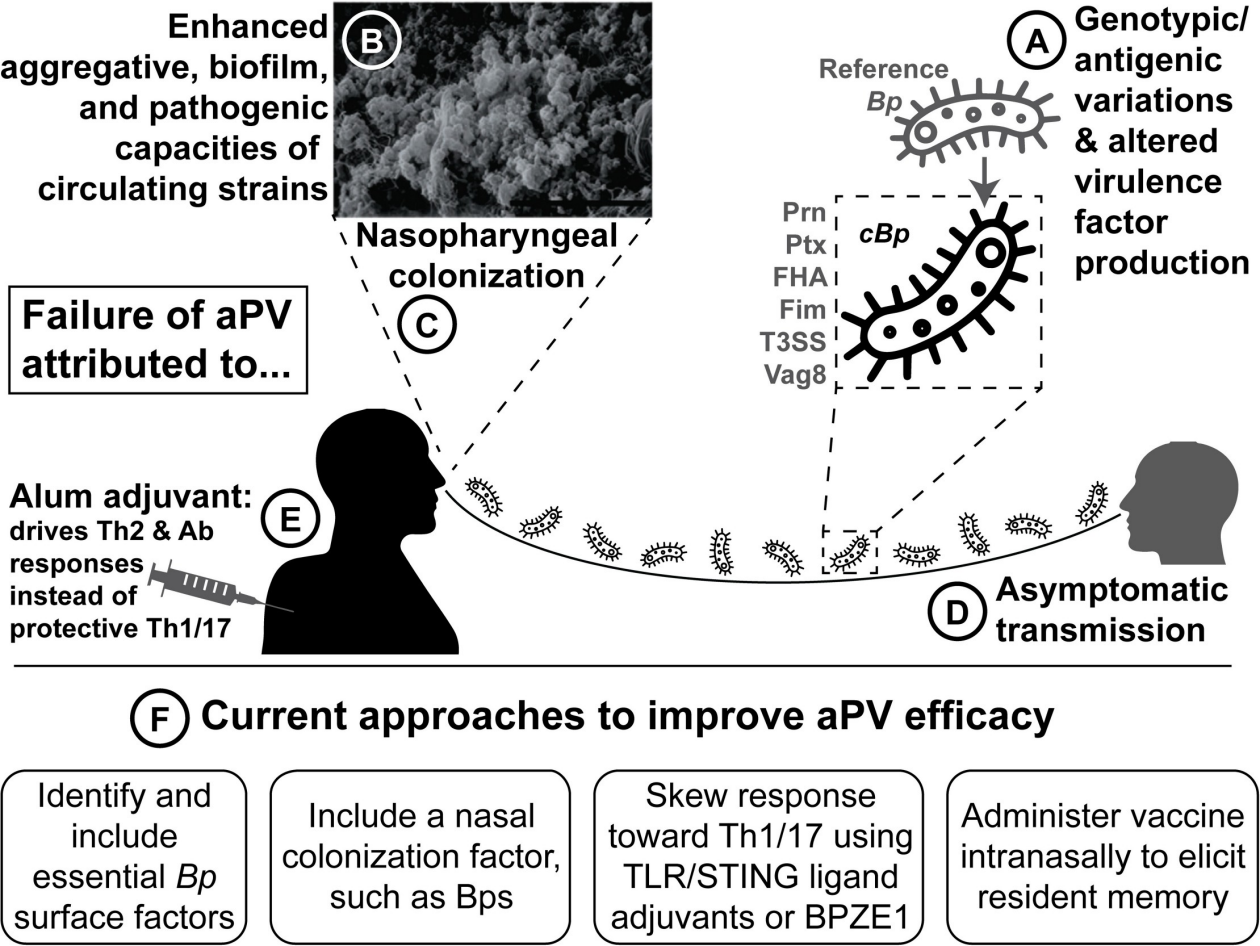
Reasons for and research directions to control pertussis resurgence. Resurgence of aPV is attributed to (A) genotypic and antigenic variation and the differential production of virulence factors between currently circulating strains (CBp) and the reference strains; (B) increased aggregation, biofilm formation, adhesion, and colonization phenotypes of CBp strains; (C) inability to reduce nasopharyngeal colonization; (D) subsequent transmission; and (E) suboptimal immune response induced by alum, an adjuvant in aPV which drives Th2 and antibody responses instead of the Th1/17 responses required for protection of the respiratory tract. (F) Multiple approaches are suggested with the goal of developing novel and more effective next-generation aPV. aPV, acellular pertussis vaccine; Bp, *Bordetella pertussis*; cBp, circulating *Bp*.

As described above and summarized in Fig 1, future research must address (1) the phenotypic and pathogenic differences in *cBp* strains; (2) the failure of aPV to prevent nasopharyngeal colonization of *Bp* and subsequent transmission, and (3) the suboptimal and short-lived duration of aPV-induced protection. To address these issues, research efforts should prioritize (1) inclusion in the aPV of conserved and essential antigens and those involved in the colonization of nasopharynx; (2) testing the efficacy of mucosal immunization and understanding the underlying vaccine-elicited immunological responses; (2) inclusion of improved Th1/17-skewing adjuvants, and (4) the development and use of in vitro and ex vivo systems that mimic the human respiratory tract environment, thereby improving the understanding of host–pathogen interactions in the context of human disease. Together, these strategies will lead to more effective next-generation vaccines that will protect against this highly contagious human pathogen.
